# The First Asian, Single-Center Experience of Blastocyst Preimplantation Genetic Diagnosis with HLA Matching in Thailand for the Prevention of Thalassemia and Subsequent Curative Hematopoietic Stem Cell Transplantation of Twelve Affected Siblings

**DOI:** 10.1155/2020/5292090

**Published:** 2020-06-26

**Authors:** Kasorn Tiewsiri, Somjate Manipalviratn, Warachaya Sutheesophon, Preeda Vanichsetakul, Piyarat Thaijaroen, Pagawadee Ketcharoon, Cara K. Bradley, Steven J. McArthur, Weena Krutsawad, James T. A. Marshall, Konstantinos I. Papadopoulos

**Affiliations:** ^1^Superior A.R.T., 1126/2 Vanit Building II, New Petchburi Road, Makkasan, Ratchathewi, Bangkok 10400, Thailand; ^2^THAI StemLife, 566/3 Soi Ramkhamhaeng 39 (Thepleela 1), Prachaouthit Rd., Wangthonglang, Wangthonglang, Bangkok 10310, Thailand; ^3^Hematopoietic Stem Cell Transplant Unit, Wattanosoth Hospital, 2 Soi Soonvijai 7, New Petchburi Road, Huaykwang, Bangkok 10310, Thailand; ^4^Genea, 321 Kent Street, Sydney, New South Wales 2000, Australia

## Abstract

**Results:**

In 221 cycles from 138 patients (104 cycles requiring HLA matching), 90.5% had embryo(s) biopsied for genetic testing. There were 119 embryo transfers for thalassemia (76) and thalassemia-HLA cases (43), respectively, resulting in overall clinical pregnancy rates of 54.6%, implantation rates of 45.7%, and live birth rates of 44.1%. Our dataset included fifteen PGD-HLA live births with successful HSCT in twelve affected siblings, 67% using umbilical cord blood stem cells (UCBSC) as the only SC source.

**Conclusions:**

We report favorable thalassemia PGD and PGD-HLA laboratory and clinical outcomes from a single center. The ultimate success in PGD-HLA is of course the cure of a thalassemia-affected sibling by HSCT. Our PGD-HLA HSCT series is the first and largest performed entirely in Asia with twelve successful and two pending cures and predominant UCBSC use.

## 1. Introduction

Thalassemia is the most common monogenic disease worldwide [1]. Several clinical forms of *α*-thalassemia and *β*-thalassemia have been described and are considered a public health issue [1]. Various mutations in the genes coding for the *α*- or *β*-globin subunits cause absent or reduced production of hemoglobin subunits resulting in imbalance of the *α*/*β*-globin chain ratio, chronic hemolysis, and ineffective erythropoiesis [1]. The patients require long-term regular red blood cell transfusions and chelation therapy for the resulting iron overload [1]. As the human body has no means to excrete iron, iron overload subsequently leads to multiorgan dysfunction (particularly the liver, heart, anterior pituitary, and pancreatic *β*-cells), the hallmark of clinical morbidity in thalassemia [1]. Children with untreated or partially treated *β*-thalassemia die in the first or second decade of life [1]. An estimated 1.7% of the world's population are heterozygous for *α*- or *β*-globin variants, and a minimum of 0.44 per 1000 births are affected by thalassemia, with the prevalence higher in the tropical and subtropical regions of the world as carriers or mild forms of thalassemia offer some protection from malaria [1, 2]. This includes Southeast Asia, where 0.66 per 1000 conceptions have been conservatively estimated to be affected by thalassemia [3–6]. Cure is only possible by hematopoietic stem cell transplantation (HSCT) with stem cells from umbilical cord blood (UCB), peripheral blood (PB), or bone marrow (BM) from a human leukocyte antigen- (HLA-) matched donor [1].

Preimplantation genetic diagnosis (PGD) is the genetic screening of embryos created in vitro by assisted conception, allowing the identification and exclusion of embryos carrying familial monogenic diseases [7]. Over the past decade, PGD has become the preferred method to prevent the transmission of familial monogenic diseases to offspring for many couples. It has been used to test embryos for hundreds of different familial monogenic diseases, including thalassemia in Southeast Asian and Eastern Mediterranean patients where these conditions are most prevalent [7–9]. Couples with either a history of pregnancy termination after prenatal diagnosis or those who know their at-risk genetic status but who do not accept the risk of and do not wish to endure the dilemma of a probable premature termination can be offered this procedure [10]. Additionally, those couples with a disease-affected child can be offered this procedure to screen for nonaffected, HLA-compatible embryos to their disease-affected child [7, 10]. Ideally, following successful implantation of an HLA-compatible embryo and completed gestation, the birth of such a child will allow for UCB collection for a subsequent fully matched related allogeneic UCB hematopoietic stem cell transplantation (UCB-HSCT) of the affected sibling [10]. Ultimately, in the best-case scenario, at least two unaffected and healthy children will be bestowed upon such families. PGD is most commonly performed by biopsy of blastomeres from day 3 cleavage stage embryos or by biopsy of herniating trophectoderm from day 5 or 6 hatching blastocysts, with the latter now considered to be the safest method [11]. In addition to testing of biopsies for the genetic mutation, in more recent years, PGD programs have adopted comprehensive chromosome screening (CCS) using comparative genomic hybridization (CGH) and next-generation sequencing (NGS) to increase the chances of pregnancy [12].

Thalassemia is the most common genetic disorder in Thailand, with an estimated 30-40% of the population being mutation carriers [13]. Genetic screening in Thailand to identify carriers was established over three decades ago [13], and PGD to prevent the conception of thalassemia-affected children has been available since 2007 at our center. The first PGD-HLA successful HSCT performed entirely in the Far East and Thailand is included in this dataset [14]. In this study, we analyzed the outcomes of more than a decade of blastocyst PGD and PGD-HLA diagnostic and clinical services for the prevention and treatment of *α*- and *β*-thalassemia at our assisted conception clinic based in Bangkok, Thailand.

## 2. Materials and Methods

### 2.1. Study Design

The outcomes of consecutive assisted conception patients seeking PGD for thalassemia at Superior A.R.T. (Thailand) between 2007 and 2016 were analyzed. The first oocyte collection for the dataset was performed on April 19, 2007, and the last on November 18, 2016, while the first embryo transfer was made on April 25, 2007, and the last on January 16, 2017. The dataset included both the *α*- and *β*-thalassemia screening cycles, some of which also had HLA matching with an affected sibling and/or screening of chromosome content. Note that cases screening for HbE/*β*-thalassemia disease were included under *β*-thalassemia cases. There were no gamete donor-recipient cases in the dataset.

### 2.2. Ethics Statement

All patient couples were informed and extensively counseled by our resident multidisciplinary staff of geneticists, embryologists, and clinicians regarding relevant genetic facts pertaining to thalassemia and were provided necessary information about the PGD procedures, success rate, limitations, and possibility of misdiagnosis inherent to techniques prior to initiating IVF treatment, embryo biopsy genetic analysis, and transfer procedures. All patient couples provided written informed consent concerning IVF, PGD, and related procedures as well as the fate of the nontransferred embryos. Patients were informed about the possibilities of misdiagnosis and the cancelation of embryo transfer in the absence of HLA-matched embryos. Upon provision of the above prerequisites, ethical approval for this retrospective cohort study using deidentified patient clinical data was sought and consequently granted by Superior A.R.T.'s institutional review board in August 2017. Written informed consent prior to HSCT was part of the conventional patient care for thalassemia/HSCT and was obtained from the parents or legal guardians of minors on a case to case basis at the different departments of hematology, according to their guidelines for HSCT.

### 2.3. General Assisted Reproduction Procedures

Controlled ovarian stimulation was typically performed using a GnRH antagonist protocol with rFSH (follitropin alpha, beta) and/or hMG (menotropin) or alternatively using a GnRH long or short agonist protocol with drugs rFSH (follitropin alpha, beta) and/or hMG (menotropin). Follicle number and size was monitored by ultrasound, and once three follicles of ≥18 mm were observed, a human chorionic gonadotropin (hCG) trigger was administered with oocyte retrieval performed 35-36 h later. Collected oocytes were fertilized by ICSI, and embryos were cultured as described previously [15], with the exception that either Nunclon Delta Treated 4-Well IVF Dishes (NUNC; used up until July 2013) or 35 mm TC-Treated Cell Culture Dishes (Falcon; used from August 2013 onwards) were used. Subsequent to blastocyst biopsy and genetic screening, suitable embryos were transferred to the patient's uterus in either a fresh or a frozen-thawed cycle using a catheter (Soft-Trans Embryo Transfer Catheter Set; Cook Medical) with ultrasound guidance. For frozen-thawed cycles, either artificial cycles using estradiol (Progynova) for endometrial preparation or natural cycles, where an LH surge was monitored in patient blood and ultrasound scan was used to determine appropriate timing for embryo transfer, were performed. After embryo transfer, vaginal progesterone (Crinone, Cyclogest, or Utrogestan) was used until the *β*-hCG pregnancy blood test approximately ten days later; patients who were not pregnant ceased vaginal progesterone, and pregnant patients continued its use until 7 to 10 weeks gestation.

### 2.4. Blastocyst Biopsy

Blastocyst biopsy was performed essentially as described previously [16]. In brief, embryos underwent assisted hatching on day 3 or day 4 by creating a 15 *μ*m opening in the zona pellucida using a laser (ZILOS-tk or LYKOS; Hamilton Thorne). On the morning of day 5, embryos were assessed for the presence of herniating trophectoderm. Blastocysts suitable for biopsy were secured with a glass holding pipette, the herniating trophectoderm was drawn into a biopsy pipette, and a near-infrared laser was used to separate typically five to six cells from the embryo. Those not suitable for biopsy were cultured further and reassessed on the afternoon of day 5 or on day 6 of development, and if suitable biopsied. After biopsy, the specimens were washed and immediately frozen at -20°C for later processing or DNA extraction either as described previously for samples without CGH analysis (PCR testing only) [16] or using the SurePlex DNA Amplification System for samples with array-CGH-based PGS. Note that all required genetic tests were performed using a single trophectoderm biopsy; embryos whose biopsies failed to produce test results were rebiopsied if possible, which was typically performed on the same culture day as the original biopsy.

### 2.5. Thalassemia Testing

Thalassemia screening of biopsied trophectoderm cells was performed by PCR amplification of linked polymorphisms (short tandem repeat (STR) markers near the thalassemia mutation) and analysis of amplicon size. For each embryo, 3 to 5 informative STR markers were compared with results from peripheral blood samples of the mother, father, affected sibling (if applicable), and grandparents (if available). With the exception of some *α*-thalassemia mutations, embryos also had either sequencing of the patient-specific mutation or, for some thalassemia deletion mutations, screening by PCR amplification of the deleted region and amplicon size analysis. In all cases, a multiplex PCR reaction (including primers for linkage analysis and primers for testing of thalassemia mutation) was performed as described in detail previously [16] with the following exceptions: a Mastercycler ep Gradient S (Eppendorf) thermocycler was used; FastStart™ Taq DNA Polymerase (Sigma-Aldrich) was used for primary PCR reactions; Phusion High-Fidelity PCR Master Mix (Thermo Fisher) with heminested primers and 33 PCR cycles was used for secondary PCR reactions intended for mutation sequencing. Amplicon size analysis of PCR products (linkage analysis and thalassemia deletion mutations) was performed as described previously [16] with the exception that capillary electrophoresis was conducted with the ABI 3130 Genetic Analyzer (Applied Biosystems) with analysis using GeneMapper ID Software version 3.2 (Applied Biosystems). Sequencing of PCR reactions for thalassemia mutations was performed as described previously [16].

All embryos were screened for thalassemia mutations with the exception of embryos found to be abnormal by CCS using CGH. Hundreds of gene mutations to hemoglobin subunits are known resulting in complicated genetics and phenotype correlations (reviewed in [4, 5]). In general, *α*-thalassemia carriers are silent carriers (mutation to 1 of 4 alleles) or those with *α*-thalassemia trait (mutation to 2 of 4 alleles); note that *α*-thalassemia carrier embryos were preferentially transferred over those with *α*-thalassemia trait and that, in a few cases, patients opted not to transfer embryos with *α*-thalassemia trait. Alpha-thalassemia-affected patients are those with HbH disease (mutation to 3 of 4 alleles) and *α*-thalassemia major (mutation to all 4 alleles). Beta-thalassemia carriers are carriers with mutation of one allele (B+/B or B0/B), some of which may present with thalassemia minor and the heterozygous state for HbE. Additionally, patients with homozygous HbE alleles, which typically present as normal or with minor thalassemia, were classified as *β*-thalassemia carriers, with homozygous HbE embryos considered suitable to transfer although normal or heterozygous HbE embryos were transferred preferentially. Beta-thalassemia-affected patients are those with thalassemia major (B0/B0), thalassemia intermedia (B0/B+ or B+/B+), or HbE/*β*-thalassemia (compound heterozygosity for the HbE and *β*-thalassemia mutations). Note that there were no cases with autosomal dominant *β*-thalassemia, compound heterozygosity for *α*- and *β*-globin mutations, delta-beta-thalassemia, or screening only for HbE disease.

### 2.6. HLA Haplotype Analysis

Embryos requiring haplotyping to ensure HLA histocompatibility with a thalassemia-affected sibling underwent HLA haplotype matching by linkage analysis using a panel of STR markers for the MHC region on chromosome 6, with comparison to results from the mother, father, affected child, and, if available, grandparents. This was performed by multiplex PCR as per thalassemia testing, as described above. For each family, at least 6 informative and/or semi-informative STR markers were selected for HLA matching to ensure the presence of enough markers for HLA matching with the disease-affected sibling and to identify monosomy, trisomy, recombination, allelic dropout, and uniparental disomy of the HLA regions.

### 2.7. Analysis of Chromosome Composition

A proportion of stimulation cycles were also designed for chromosome screening, either PCR analysis of chromosome 21 content or CCS by CGH. Embryo screening for chromosome 21 content was performed simultaneously with thalassemia screening from 2011 onwards for a small number of cycles. At least three informative STR markers on chromosome 21 were examined to ensure the presence of enough markers to aid the identification of aneuploidy. This was performed as described above for thalassemia testing. In 2014, CCS by CGH was adopted for the majority of PGD patients screened for monogenic disorders regardless of whether HLA matching was required. CGH was performed prior to thalassemia screening, and only embryos with no detectable anomalies were subsequently tested for thalassemia mutations and, if required, HLA typing. CGH was performed on embryo biopsies using the SurePlex DNA Amplification System (whole genome amplification kit) and the BlueGnome 24sure BAC array (Illumina) as per the manufacturer's instructions.

### 2.8. Blastocyst Vitrification and Warming

Between years 2007 and 2010, blastocysts were cryopreserved by vitrification using solutions from the Sydney IVF Blastocyst Vitrification Kit (Cook Medical) with the CVM Vitrification Kit including CVM Fibreplug device (Cryologic) according to the manufacturer's instructions. Between 2011 and 2016, blastocysts were vitrified using VT601 Kitazato vitrification media and open Cryotop device (Kitazato) according to the manufacturer's instructions. Warming of vitrified blastocysts was performed using either the Sydney IVF Blastocyst Warming Kit (Cook Medical; used between 2007 and 2010) or the VT602 Kitazato thawing media (Kitazato; used between 2011 and 2016) according to the manufacturer's instructions. After warming, embryos were cultured in the blastocyst medium for approximately 2 h before assessment of survival of the vitrification-warming process; in general, a vitrified-warmed embryo was considered to have survived and thus be suitable for uterine transfer if greater than 50% of cells were intact with some observable reexpansion.

### 2.9. UCB Collection and Stem Cell Processing

UCB was collected from PGD-HLA births in preparation for HSCT of an affected sibling. In all cases, cord blood was collected in utero by gravity from an umbilical vein by the THAI StemLife physician attending the delivery, using a sterile THAI StemLife-provided personal cord blood collection kit including a 21 G needle and a 250 mL labeled blood collection bag containing 35 mL of citrate phosphate dextrose anticoagulant, stored at 15°C to 25°C in a temperature-validated shipper containing a temperature log kit, and transported to the THAI StemLife processing laboratory to be processed immediately after arrival. The process was performed either manually or using the automated SEPAX system. The manual process includes red blood cell depletion after Hemohes addition, plasma reduction, and volume depletion after double-refrigerated centrifugation. White blood cell (WBC) and total nucleated cell (TNC) counts were performed with automated cell counter (Coulter AcT5 Diff), as were CD34+ (Coulter flow cytometer Epics XL), recovery rates, and viability by Trypan blue or flow cytometry via Coulter flow cytometer Epics XL, before postprocess infectious testing and DMSO addition. Subsequently, after transfer to a thermogenesis and overlap bags, controlled rate freezing ensued to −180°C before the final storage in liquid nitrogen at −196°C according to international standards [17]. Frozen samples were subsequently retrieved upon request and transferred in a cryoshipper to the desired HSCT hospital centers where they were thawed by the THAI StemLife laboratory staff at the discretion of the treating hematologist.

### 2.10. Hematopoietic Stem Cell Transplantation (HSCT)

Our HSCT procedures complied with worldwide standard practice guidelines and recommendations. Although bone marrow is considered to be the first-choice source of hematopoietic cells from HLA-identical siblings, UBC can be also considered from newborn siblings if feasible [18]. A double-lumen central venous (Hickman) catheter was placed in every recipient/patient prior to initiating the pretransplant myeloablative conditioning regimen. Pretransplant conditioning chemotherapy composed of busulfan (Bu) 4-4.8 mg/m^2^/day for 4 days plus fludarabine (Flu) 35 mg/m^2^/day for 5 days plus rabbit antithymocyte globulin (rATG) of 1, 2, and 3 mg/kg/day for 3 consecutive days, respectively [19, 20]. These preparative regimens usually took 8 days and were completed at least 1 day before infusion day (transplant day or day zero). On transplant day, the UCB unit which had been identified would be transported to the transplant unit under cryogenic conditions in a portable cryoshipper from the THAI StemLife storage site and would be thawed just before the infusion took place through the Hickman catheter into the recipient's blood circulation. During the pre- and early posttransplant period, prophylactic antimicrobial medications were provided. Protective environmental precautions were applied. The recipient had to remain in an isolated, positive-pressure, high-efficiency particulate aerosol (HEPA) filtration room. All cellular blood components, such as packed red cell units and platelet apheresis units, were leukocyte-filtered and irradiated before being supportively transfused [21]. After completion of UCB infusion, graft-versus-host disease (GvHD) prophylaxis with continuous intravenous cyclosporine would be administered. Every patient was given recombinant human granulocyte colony-stimulating factor (G-CSF) after transplantation, infused intravenously once daily until the neutrophil count exceeded 2 × 10^9^/L for 2 consecutive days, which usually happened in 14-21 days posttransplant, when G-CSF would be discontinued.

### 2.11. Assisted Conception Outcome Measures

The stimulation cycle number, maternal and paternal age (Table 1), and number of embryos transferred per transfer (Table 2) were presented as median with interquartile range due to a skewed distribution. Note that the maternal and paternal age was recorded in years for each cycle at the time of oocyte collection. The clinical pregnancy rate was the number of patients with an intrauterine fetal heart-positive pregnancy (fetal heart motion as detected by ultrasound at 6 to 7 weeks of pregnancy; note that some cases had screening up to 12 weeks of pregnancy) per embryo transfers. The implantation rate was the number of fetal hearts per embryos transferred. The live birth rate was the number of patients with a clinical pregnancy that had a live birth per the number of patients with a clinical pregnancy for whom live birth data was known. The multiple birth rate was the number of multiple births per live births. The miscarriage rate was the number of patients with a clinical pregnancy that did not result in a live birth per the number of patients with a clinical pregnancy for whom live birth data was known.

### 2.12. HSCT Outcome Measures

Neutrophil engraftment was defined as the first day an absolute neutrophil count (ANC) rose over 0.5 × 10^9^/L for 3 consecutive days. Platelet recovery was defined as the first day of a platelet count over 20 × 10^9^/L for 3 consecutive days without platelet transfusion support. Graft failure or rejection was defined if ANC did not rise or no neutrophil engraftment within 28 days after HSCT or ANC declined below 0.2 × 10^9^/L after initial recovery. After neutrophil engraftment, acute GvHD, if any, would be diagnosed and graded according to previously reported criteria [22]. Engraftment evaluations were studied by chimerism analysis using the microsatellite, short tandem repeat method. For cases of gender mismatch between recipients and donors, the techniques of fluorescent in situ hybridization (FISH) of the XX-XY chromosome were also used. Complete donor engraftment was defined as 100% of donor cells. Stable mixed-chimera states with donor predominance (>95% belonging to donor cells) were also defined as satisfactory engraftment. Any of the above states, along with the absence of GvHD occurrence, were defined as successful outcomes.

## 3. Results

### 3.1. Family History, Indications, and Patient Origin

This study analyzed the outcomes of a decade of PGD for the prevention and treatment of *α*- and *β*-thalassemia. There were 138 patients who underwent assisted conception for thalassemia screening, 50 (36.2%) of which also had HLA matching for the potential treatment of a thalassemia-affected sibling (Table 1). These patients underwent a total of 221 stimulation cycles, with a median maternal and paternal age of 34.0 and 36.3 years, respectively. For the vast majority of cycles, both parents were carriers of thalassemia (205; 92.8%), with the remaining cycles having one thalassemia-affected parent and the other a carrier of the disease (16; 7.2%). Most of the patients were from Thailand (60; 43.5%), where the clinic is located, with the remaining cycles mostly from nearby countries including China (32; 23.2%), India (19; 13.8%, of which 79% sought PGD-HLA services), and Vietnam (13; 9.4%).

### 3.2. Embryo Genotyping Outcomes

Of the 221 stimulation cycles for thalassemia screening, 52 (23.5%) were also scheduled for CCS using CGH and 7 (3.2%) for chromosome 21 analysis by PCR (Table 3). Analysis of screening outcomes revealed that 90.5% (200/221) of cycles achieved a blastocyst biopsy and 71.9% (159/221) of cycles had at least one screened embryo suitable for uterine transfer. The proportion of biopsied cycles with at least one screened embryo suitable for transfer was higher for cycles with thalassemia screening than thalassemia screening plus HLA matching: 94.3% (99/105) and 63.2% (60/95), respectively. Overall, there were 1180 blastocysts biopsied, with 58.6% (343/585) of thalassemia and 16.8% (100/595) of thalassemia-HLA embryos suitable for clinical use. Only a small proportion (21/1180; 1.8%) of biopsied embryos failed to achieve a result for thalassemia screening and/or HLA matching.

### 3.3. Embryo Transfer Outcomes

There were 119 embryo transfers, of which 51 (42.9%) were performed as fresh cycles while 68 (57.1%) were frozen cycle embryo transfers, with a total of 164 blastocysts transferred (Table 2). Regarding the frozen embryo transfers, 110 vitrified embryos were warmed with intent for transfer, 10 (9.1%) of which were not transferred as they were not recovered from the vitrification device or were considered not to have survived the vitrification-warming process. The data revealed an overall clinical pregnancy rate of 54.6% (65/119) and implantation rate of 45.7% (75/164). The pregnancy rate for *α*- and *β*-thalassemia cases and *α*- and *β*-thalassemia-HLA cases was 57.9% (44/76) and 48.8% (21/43), respectively. After taking into consideration the number of embryos transferred, the implantation rate for thalassemia and thalassemia-HLA cases was 45.2% (52/115) and 46.9% (23/49), respectively. Combined, the live birth rate was 44.1% (52/118; 1 with missing data) which included 15 live births (15/43; 34.9%) (one of which was a twin birth) of babies with thalassemia plus HLA matching with a disease-affected sibling.

### 3.4. HSCT Outcomes

Of these 15 PGD-HLA live births, their HLA-compatible hematopoietic stem cells were used in twelve successful HSCT (eight girls and four boys). In eight cases, UCB stem cells only were adequate (in one originating from identical twin siblings); three involved a combined UCB and BM transplantation from the HLA-identical sibling donors; and in one, only BM was used as the UCB sample was stored in Thailand while the HSCT took place in India (Table 4). Eight of the twelve cases were performed by a single hematologist in Thailand [14] while two cases were performed in India and one each in Thailand and China. Follow-up was available in all cases, with mild veno-occlusive disease (VOD) in two that subsequently completely resolved in both by conservative treatment, a resolved cytomegalovirus (CMV) reactivation in one, and no complications in ten. The median age for HSCT was 59.5 months while the four BM donations occurred at 12, 29, 36, and 76 months. Focusing on the case series (*n* = 8) by a single hematologist in Thailand, seven of which used UCB as a sole source of HSC and one along with BM, initial hematopoietic recovery by day 30 equaled 100% (eight out of eight patients). In terms of engraftment, no patients experienced graft failure. Five patients achieved complete donor engraftment, three patients developed stable mixed-chimera states with donor predominance, and eventually all of them were no longer blood transfusion-dependent nor required further transfusions. Their hemoglobin levels increased to the level of their corresponding donors' status. One patient developed acute grade 2 graft-versus-host disease (GvHD), possibly due to the use of BMSC and the discontinuation of cyclosporine during the mild VOD that ultimately resolved. The GvHD is currently controlled on cyclosporine and prednisolone and regressing, and the ex-patient has been released to home. None of the remaining patients (*n* = 11) have developed chronic GvHD. The overall transplant-related mortality was zero. To date, overall (OS) and disease-free survival (DFS) are both 100%. Two additional affected siblings await HSCT at a later donor age, due to financial reasons.

## 4. Discussion

The present retrospective analysis presents the outcomes of a decade of PGD and PGD-HLA diagnostic and clinical services for *α*- and *β*-thalassemia in 138 consecutive patients (221 stimulation cycles) in a single center in Bangkok, Thailand, Asia. Thalassemia is the most common monogenic disease worldwide [1, 3, 4, 6], with the rampant prevalence in South and South East Asia including Thailand, where 30-40% of the population are estimated being mutation carriers [13]. These disorders present a significant public health impact [23, 24]. Overall, we report favorable outcomes for these patients, with 90.5% of stimulation cycles achieving at least one blastocyst biopsy. Furthermore, for thalassemia and thalassemia-HLA cycles, respectively, 94.3% and 63.2% of biopsied cycles had at least one embryo suitable for clinical use, and 49.3% and 34.9% of embryo transfers resulted in a live birth.

Our live birth rates for PGD and PGD-HLA cases reported here are among the highest to date in the literature. The ESHRE PGD Consortium reported a clinical pregnancy rate of 34.3% [25] from 598 embryo transfers, up from 29% from 3727 embryo transfers occurring from 1997 to 2007 using embryos screened for almost 200 single gene disorders [8], while Kuliev et al. [9] reported a pregnancy rate of 48% from 2698 embryo transfers using 4902 embryos screened for single gene disorders including HLA cases. Additionally, Kahraman et al. [10] reported a live birth rate of 28.3% from 212 embryo transfers using embryos screened for inherited mutations and HLA matching with a disease-affected sibling requiring HSCT. Our results add further to the evidence that patients undergoing PGD for monogenic disorders, including those with HLA matching, have a favorable chance of a live birth when embryos are available for transfer.

Of the 138 couples who attended our clinic for PGD for thalassemia, 50 (36.2%) had a thalassemia-affected child (48 with *β*-thalassemia) and were seeking HLA matching in addition to thalassemia screening. To date, there have been 15 live births, of which the HLA-compatible hematopoietic stem cells of twelve have been used for successful, curative, uncomplicated HSCT of the disease-affected siblings (UCB only in eight, combined UCB and BM transplantation in three, and BM only in one), which is of course the measure of the true clinical efficacy of PGD-HLA. Two additional affected siblings await HSCT at a later donor age due to reasons not involving UCB stem cell numbers. The present series includes the Far East's first successful PGD-HLA HSCT [14] and is to date the largest series performed in Asia (outside Turkey) in its entirety. In the eight HSCT cases where UCB only was used, the collected samples contained enough HSC to successfully reconstitute the recipients' lymphohematopoietic compartment proving that in the hands of experienced personnel, UCB offers unique advantages such as easy and painless procurement. All our twelve patients were engrafted successfully (minor mostly self-resolving complications were recorded in two out of twelve cases), possibly related to an early median age of HSCT at 59.5 months. Not all HSCT cases are successful with graft failure reported in four of 37 transplants by Kahraman et al. [10], all of which used combined UCB and BM, possibly related to an older age at HSCT and large number of blood transfusions in patient history. Published HSCT series clearly document that when HSC are needed in hemoglobinopathies, a related allogeneic source is seen in more than 86% of the cases [26]. Thus, at present, PGD with HLA typing offers the most realistic chance for cure in hemoglobinopathies as it will provide HLA-identical UCB or BM HSC from an HLA-identical sibling ensuring excellent treatment outcomes [7, 25, 27]. In the future, correcting pathogenic gene mutations in human embryos [28] and *β*-thalassemia using base editing in human somatic cells and embryos [29] may offer other valid alternatives.

Similar HLA typing without mutation analysis has been performed in acquired diseases such as acute myeloid leukemia and acute lymphoid leukemia, where allogeneic HSCT is required from an HLA-identical donor to cure the disease [25]. To date, PGD has expanded to testing for nearly 200 conditions including late-onset disorders with genetic predispositions [25, 30], a long way since the first PGD-HLA typing for Fanconi anemia in 2001 [31]. Moreover, PGD can be used to identify cancer predispositions as ataxia-telangiectasia, familial adenomatous polyposis, familial colorectal cancer, breast cancer (BRCA1 and BRCA2), von Hippel-Lindau syndrome, retinoblastoma, neurofibromatosis types 1 and 2, Gorlin syndrome, tuberous sclerosis, and familial posterior fossa brain tumors [32].

Despite the optimistic outlook, PGD with HLA typing is not an easy endeavor [33]. A cross-disciplinary approach is needed in a specialized PGD center where reproductive endocrinologists, embryologists, molecular biologists, and geneticists coordinate and interact with experienced obstetricians, an umbilical cord blood banking facility with prior successful cord blood sample releases, and a specialist hematology department to seamlessly facilitate the procedure. Limitations in the success of this strategy must be made clear to the parents [10]. Limitations related to UCB-HSCT include the limited number of collected UCB stem cells relative to recipient weight, but this can be overcome with delaying the HSCT and combining available UCB stem cells with harvested BM stem cells from the donor sibling at a later age as seen in the present series.

In conclusion, this retrospective, decade-long, single Thai center study demonstrates favorable thalassemia PGD and PGD-HLA outcomes pointing out that Asia has come of age in providing PGD and PGD-HLA diagnostic and clinical services for the prevention and treatment of thalassemia. PGD has the potential to reduce thalassemia-related abortions and births in South and South East Asia while PGH-HLA offers the only immediately available and realistic strategy for the radical cure of thalassemia-affected patients. The ultimate success in PGD-HLA is of course the cure and full recovery of a thalassemia-affected sibling by HSCT. Our PGD-HLA HSCT series is the first and largest performed entirely in Asia with twelve successful and two pending cures predominantly using UCB.

## Figures and Tables

**Table 1:** Demographics of patients that underwent PGD and PGD-HLA for thalassemia.

	*α*-Thalassemia	*α*-Thalassemia-HLA	*β*-Thalassemia	*β*-Thalassemia-HLA	Total
Patients	46	2	42	48	138
Stimulation cycles					
Total	57	2	60	102	221
Cycle number^a^	1 (1-1)	1 (n/a)	1 (1-2)	2 (1-3)	1 (1-2)
Age^a^					
Maternal	34.3 (30.3-37.1)	34.1 (n/a)	33.6 (31.3-36.6)	33.8 (30.9-36.6)	34.0 (30.9-36.7)
Paternal	36.1 (32.1-40.6)	39.0 (n/a)	35.7 (33.4-39.3)	36.5 (33.8-40.7)	36.3 (33.3-40.0)
Genetic status^b^					
Carrier/carrier	51	2	59	93	205
Affected/carrier	6	0	1	9	16
Country					
Southeast Asia					
Thailand	16	1	26	17	60
Vietnam	6	1	3	3	13
Cambodia	1	—	—	3	4
Malaysia	—	—	—	4	4
Laos	1	—	—	—	1
South Asia					
India	—	—	4	15	19
Maldives	—	—	2	—	2
Nepal	—	—	—	1	1
East Asia					
China	22	—	7	3	32
Other					
UAE	—	—	—	2	2

^a^Median (IQR). ^b^Alpha-thalassemia carriers are either silent carriers or those with *α*-thalassemia trait. Live-born alpha-thalassemia-affected patients are those with HbH disease. Beta-thalassemia carriers are silent carriers, those with the thalassemia minor trait, or homozygous for the HbE allele. Beta-thalassemia-affected patients are those with thalassemia major, thalassemia intermedia, or HbE/*β*-thalassemia.

**Table 2:** Clinical outcomes of embryo transfer of PGD and PGD-HLA blastocysts screened for thalassemia.

	*α*-Thalassemia	*α*-Thalassemia-HLA	*β*-Thalassemia	*β*-Thalassemia-HLA	Total
Embryo transfers	33	1	43	42	119
Fresh cycles	14 (42.4%)	0	16 (37.2%)	21 (50.0%)	51 (42.9%)
Frozen cycles	19 (57.6%)	1 (100%)	27 (62.8%)	21 (50.0%)	68 (57.1%)
Embryos vitrified-warmed	32	4^d^	49	25	110
Not recovered/survived^a^	4 (12.5%)	1 (25.0%)	4 (8.2%)	1 (4.0%)	10 (9.1%)
Recovered and survived	28 (87.5%)	3 (75.0%)	45 (91.8%)	24 (96.0%)	100 (90.0%)
Embryos transferred	52	3	63	46	164
Embryos per transfer^b^	2 (1-2)	3 (n/a)	1 (1-2)	1 (1-1)	1 (1-2)
Outcomes^c^					
Clinical pregnancies	19/33 (57.6%)	1/1 (100%)	25/43 (58.1%)	20/42 (47.6%)	65/119 (54.6%)
Implantations	21/52 (40.4%)	1/3 (33.3%)	31/63 (49.2%)	22/46 (47.8%)	75/164 (45.7%)
Live births	17/32 (53.1%)^e^	1/1 (100%)	20/43 (46.5%)	14/42 (33.3%)	52/118 (44.1%)^e^
Multiple births	2/17 (11.8%)	0/1 (0%)	4/20 (20.0%)	1/14 (7.1%)	7/52 (13.5%)
Clinical miscarriages	1/18 (5.6%)^e^	0/1 (0%)	5/25 (20.0%)	6/20 (30%)	12/64 (18.8%)^e^

^a^Embryos not recovered from the vitrification device upon warming or considered not to have survived the vitrification-warming process. ^b^Median (IQR). ^c^Assessed by ultrasound typically at 6 to 7 weeks of pregnancy (up to 12 weeks for some cases) and defined by the presence of a fetal heartbeat. Note there was one ectopic pregnancy from the *α*-thalassemia group (not considered a clinical pregnancy). ^d^Three embryos were warmed from one patient and one embryo from another patient, the latter of which did not survive the vitrification-warming process, and thus, this patient did not have an embryo transfer. ^e^Outcome (birth or miscarriage) unknown for one *α*-thalassemia case with a clinical pregnancy.

**Table 3:** PGD results of embryos that underwent screening for thalassemia.

	*α*-Thalassemia	*α*-Thalassemia-HLA	*β*-Thalassemia	*β*-Thalassemia-HLA	Total
Stimulation cycles	57	2	60	102	221
Oocytes retrieved	791	84	925	1449	3249
Mature (%/oocyte retrieved)	635 (80.3%)	69 (82.1%)	732 (79.1%)	1178 (81.3%)	2614 (80.5%)
2PN (%/mature)	468 (73.7%)	48 (69.6%)	573 (78.3%)	911 (77.3%)	2000 (76.5%)
Additional screening^a^					
None	38 (66.7%)	2 (100%)	34 (56.7%)	88 (86.3%)	162 (73.3%)
All chromosomes (CGH)	15 (26.3%)	0	23 (38.3%)	14 (13.7%)	52 (23.5%)
Chromosome 21 (PCR)	4 (7.0%)	0	3 (5.0%)	0	7 (3.2%)
Cycles					
With embryos for biopsy	51 (89.5%)	2 (100%)	54 (90.0%)	93 (91.2%)	200 (90.5%)
With suitable embryo(s)^b^	47	2	52	58	159
With no suitable embryo(s)^b^	4	0	2	35	41
With no embryos for biopsy	6 (10.5%)	0	6 (10.0%)	9 (8.8%)	21 (9.5%)
Number of biopsied embryos	259	23	326	572	1180
Embryo screening results^c^					
Suitable	146 (56.4%)	6 (26.1%)	197 (60.4%)	94 (16.4%)	443 (37.5%)
Chromosome(s) screened	24	0	69	6	99
Chromosome(s) not screened	122	6	128	88	344
Nonsuitable	108 (41.7%)	17 (73.9%)	125 (38.4%)	466 (81.5%)	716 (60.7%)
Thalassemia-affected^d^	80	6	79	37	202
Abnormal chromosome(s)	28	3	46	55	132
HLA nonmatch	—	8	—	374	382
No result^e^	5 (1.9%)	0	4 (1.2%)	12 (2.1%)	21 (1.8%)

^a^Chromosome screening additional to thalassemia and (if appropriate) HLA testing scheduled to occur if biopsiable embryos were obtained. ^b^Suitable refers to suitability for clinical use (embryo transfer) based upon genetic screening results. ^c^For cases with additional HLA screening or chromosome 21 analysis, testing was performed simultaneously with thalassemia testing. Embryos affected by thalassemia and with abnormal chromosome 21 content were listed as thalassemia-affected. ^d^Embryos affected by thalassemia and were an HLA mismatch were listed as thalassemia-affected. For cases with additional comprehensive chromosome screening, CGH was performed first and only embryos with no detectable abnormalities were subsequently screened for thalassemia (and HLA if required). Embryos with normal chromosome content that were affected by thalassemia and were an HLA mismatch were listed as thalassemia-affected. Thalassemia-affected for *α*-thalassemia cases are those with embryos with the genetics for HbH disease or *α*-thalassemia major. Embryos with the genetics for *α*-thalassemia trait or silent carriers were considered clinically suitable. Thalassemia-affected for *β*-thalassemia cases are those embryos with the genetics for thalassemia intermedia, thalassemia major, or HbE/*β*-thalassemia. Embryos which were *β*-thalassemia carriers were considered suitable for clinical use. Embryos heterozygous or homozygous for the HbE allele were also considered clinically suitable, although the former was preferentially transferred over the latter. ^e^These embryos failed to produce a result from initial biopsy (and if performed, rebiopsy) and were not suitable for further biopsy.

**Table 4:** Details and outcomes of PGD-HLA HSCT.

	Recipient sex	PGD-HLA donor sex and Dx^a^	Patient origin	Affected sibling thalassemia mutation diagnosis	HSC source	Recipient age at HSCT (months)	HSCT status/country HSCT performed	HSCT-related complications
1	M	M (normal)	Thailand	BThal/HbE Cd.17 (A>T)/HbE	UCB	59	Success/Thailand	None
2	F	F (HbE trait)	Thailand	BThal/HbE HbE/IVSII-654 (C>T)	UCB	45	Success/Thailand	None
3	F	M (HbE trait)	Thailand	BThal/HbE Cd.41/42 (-TCTT)/HbE	UCB	67	Success/Thailand	None
4	M	F (HbE trait)	Thailand	BThal/HbE Cd.41/42 (-TCTT)/HbE	UCB	39	Success/Thailand	None
5	M	F/F (IVSI-5)	Thailand	BThal/HbE HbE/IVSI-5 (G>C)	UCB twins	56	Success/Thailand	Mild VOD, completely resolved
6	F	F (normal)	Thailand	homoB-thal IVSII-654 (C>T)/Cd.41/42 (-TCTT)	UCB	45	Success Thailand	None
7	M	M (Beta-28)	Thailand	homoB-thal beta-28 (A>G)/Cd.17 (A>T)	UCB in/ex utero collection	36	Success/Thailand	None
8	F	F (HbE trait)	Thailand	B-thal/HbE HbE/IVSII-654 (C>T)	UCB + BM^c^ (BM donor age 76 months)	202	Success/Thailand	Grade 2 acute GvHD currently regress on cyclosporine and prednisolone. Mild VOD, thrombocytopenia, and CMV reactivation, all completely resolved
9	F	M (normal)	Cambodia	B-thal/HbE HbE/Cd.17 (A>T)	BM^c^ (BM donor age 12 months) (UCB not used)	132	Success/India	None
10	M	F (normal)	Cambodia	B-thal/HbE HbE/IVSII-654 (C>T)	UCB	Awaiting HSCT		
11	F	M (normal)	China	homoB-thal beta-28/Cd.41/42 (-TCTT)	UCB + BM^c^ (BM donor age 36 months)	108	Success/China	None
12	F	M (normal)	India	homoB-thal IVSI-5 (G>C)/IVSI-1 (G>A)	UCB	60	Success/India	None
13	M	M (carrier)	India	homoB-thal IVSI-5 (G>C)/IVSI-5 (G>C)	N/A^b^	Unknown		
14	F	M (IVSI-5)	India	homoB-thal Cd.41/42 (TCTT)/IVSI-5 (G>C)	UCB + BM^c^ (BM donor age at 29 months)	75	Success/Thailand	None
15	F	F (*αα*/--^SEA^)	Vietnam	HbH-CS disease (--/*α*^Cs^*α*)	UCB	Awaiting HSCT		

^a^Dx = diagnosis. ^b^N/A = not applicable. ^c^Same donor. CMV = cytomegalovirus; VOD = veno-occlusive disease; GvHD = graft-versus-host disease.

## Data Availability

The raw deanonymized data used to support the findings of this study are available and can be released after consideration by the corresponding author. Detailed treatment data may be embargoed by the treating hospitals' and countries' patient confidentiality laws.
